# Physiological and nutritional adaptation of broodmares during the transition from late pregnancy to early lactation: Digestibility changes and the role of uNDF as an internal marker

**DOI:** 10.1007/s11259-026-11194-4

**Published:** 2026-04-09

**Authors:** Martina Lamanna, Jole Mariella, Francesca Freccero, Aliai Lanci, Riccardo Colleluori, Francesca Ghiaccio, Giovanni Buonaiuto, Emanuela Valle, Federica Raspa, Carolina Castagnetti, Damiano Cavallini

**Affiliations:** 1 Department of Veterinary Medical Sciences, University of Bologna, Ozzano dell’Emilia (BO), 40064 Italy; 2 Department of Veterinary Sciences, University of Turin, Grugliasco (TO), 10095 Italy

**Keywords:** Equine nutrition, Horses, Hindgut fermentability, Peripartum, Undigested neutral detergent fiber

## Abstract

Nutritional management of broodmares during late pregnancy and early lactation is essential for maternal health and foal well-being. This study evaluated a targeted dietary adaptation during the transition from late gestation to early lactation, monitored nutrient digestibility, and applied undigested neutral detergent fiber (uNDF) as an intrinsic digestibility marker. A prospective nutritional study was conducted on 11 clinically healthy Trotter broodmares (3 primiparous, 8 multiparous) monitored under standardized conditions. Mares received a forage-based diet with controlled starch intake and a concentrate adjusted to the physiological stage. Feed and fecal samples were collected to determine nutrient digestibility using uNDF. The dietary protocol was effective, with no evidence of impaired intestinal fermentation. Digestibility values remained within ranges reported for healthy horses (organic matter 56–63%, crude protein 69–80%, starch 79–86%, neutral detergent fiber 39–42%), suggesting no marked impairment of nutrient utilization. Based on fecal analysis, peripartum changes in fermentation were observed, particularly on the day of parturition, but remained within physiological limits. A slight, but significant, increase in fecal dry matter (+ 3%) suggested a predisposition to postpartum constipation. Starch digestibility increased during parturition (up to 87%), possibly associated with a transient reduction in gastrointestinal transit rate, while fiber digestibility remained stable. Digestibility estimates obtained using uNDF were consistent with values reported using total fecal collection, supporting its applicability as a non-invasive marker. These findings support gradual dietary transitions in broodmares and show that uNDF was successfully applied as an internal marker to verify the changes in nutrient digestibility observed during the study.

## Introduction

Inadequate or imbalanced nutrition during pregnancy and lactation can lead to significant health complications, such as metabolic disorders (e.g., obesity, insulin dysregulation) and digestive disturbances (e.g., colon impaction, colics), particularly during the peripartum period (Geor et al. 2013). Concurrently, the mare’s diet directly influences fetal development and neonatal outcomes, affecting parameters such as birth weight, foal vitality, and lactation success. Insufficient nutritional support during gestation has also been implicated in an increased risk of developmental disorders, including osteochondrosis in foals (Derisoud et al. 2021). Therefore, understanding the interplay between nutrition, digestive physiology, and health is essential to optimize management practices during these critical life stages.

Gestation in horses averages 340 days, but the duration can vary significantly (320–365 days), influencing the foal’s birth weight and the mare’s nutritional demands, particularly during peak fetal growth after the 240th day of gestation. Following parturition, lactation begins, requiring the mare to support the foal’s rapid growth, which can reach approximately 45% of its adult body weight at weaning. Milk production in broodmares ranges from 2.0 to 3.5 kg per 100 kg of body weight, equivalent to 10–17 kg/day for a 500-kg mare, with peak lactation occurring between the second and third month postpartum (Ginther 1992).

Fiber represents a cornerstone of the equine diet and primarily consists of cellulose, hemicellulose, and lignin. Laboratory methodologies allow the characterization of fiber fractions, including neutral detergent fiber (NDF), acid detergent fiber (ADF), and acid detergent lignin (ADL), which contribute to the evaluation of diet composition and potential digestibility (Saha and Pathak 2021). Apparent digestibility, defined as the difference between nutrient intake and fecal output without correction for endogenous losses, is traditionally determined through total fecal collection, a method considered accurate but labor-intensive and difficult to implement under field conditions (Schurg 1981; Bergero et al. 2009). True digestibility, in contrast, accounts for endogenous nutrient losses. To overcome practical limitations of total fecal collection, intrinsic and extrinsic markers have been proposed as alternative tools for estimating nutrient utilization in horses (Sales 2012). Indigestible fiber fractions derived from the detergent system described by Van Soest (1994) have been widely applied in ruminant research and may represent a promising approach for digestibility assessment in equine nutrition.

This study aimed to investigate three main aspects of broodmare nutrition during late pregnancy and early lactation. The first objective was to evaluate the effectiveness of a dietary adaptation protocol specifically designed for broodmares at the end of pregnancy (last two weeks), ensuring their nutritional needs are adequately met. The second focus was to monitor changes in fiber digestibility and other dietary nutrients, providing detailed insights into the physiological adaptations of the digestive system during the critical phases of late pregnancy and early lactation (two weeks before and after parturition).

In addition, as a methodological component of the study, uNDF was used as an internal marker to estimate apparent digestibility and to verify the changes occurring during the transition period.

## Materials and methods

### Ethical approval

All procedures complied with Directive 2010/63/EU on the protection of animals used for scientific purposes and adhered to the European Union’s legal standards (98/58/EC) for the welfare of farmed animals. Animal-related protocols were reviewed and approved by the University of Bologna Institutional Animal Care and Use Committee.

### Experimental design and animals

This study was designed as a prospective observational nutritional study involving client-owned mares presented for foaling management at the Equine Perinatology and Reproduction Unit (EPRU) of the Department of Veterinary Medical Sciences, University of Bologna, during the 2022 foaling season. No experimental manipulation was performed, and animals were managed according to the unit’s standard clinical routine. The mares were individually housed in stalls measuring approximately 4 × 4 m that allowed natural behaviours such as standing, lying down, and feeding (Lamanna et al. 2025a, 2025b). Each pen was daily bedded with straw and equipped with individual drinkers.

**Fig. 1:**
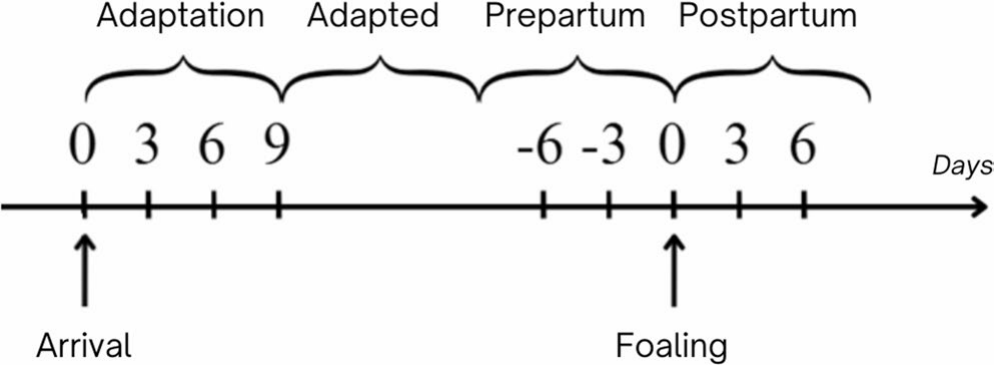
Sampling protocol, including time points and temporal intervals

The study initially enrolled 13 Trotter broodmares; however, two mares were excluded from the digestibility analysis due to the onset of postpartum constipation requiring clinical management, which could have interfered with normal digestive function, resulting in a final sample size of 11 broodmares. The cohort included three primiparous and eight multiparous mares, all with normal pregnancy. Detailed information on the mares’ signalment is provided in Table 1. The observation period extended from late gestation to early lactation until discharge and followed the standard protocol for healthy broodmares at EPRU. Depending on the parturition day, each mare had a different length of the adaptation period, with a mean duration of 15 ± 6.5 days (mean ± SD); similarly, the postpartum period varied based on the discharge date, with an average duration of 6.5 ± 2.4 days (mean ± SD) (Fig. 1). The term “adaptation period” refers to the transitional phase during which mares were progressively introduced to the dietary regimen used at EPRU.

**Table 1:** Characteristics of weight, BCS at arrival, and parity of the selected broodmares

Broodmares	Parity	Body weight, kg	BCS at arrival	Actual duration of pregnancy (days)
**n. 1**	Pluriparous	574	6.5	340
**n. 2**	Pluriparous	643	7	328
**n. 3**	Primiparous	570	6.25	338
**n. 4**	Pluriparous	550	6.5	339
**n. 5**	Pluriparous	570	6	338
**n. 6**	Pluriparous	630	7	343
**n. 7**	Pluriparous	550	6	340
**n. 8**	Primiparous	540	6	331
**n. 9**	Primiparous	500	5.5	331
**n. 10**	Pluriparous	550	6.5	325
**n. 11**	Pluriparous	540	6.5	325

BCS, Body Condition Score (scale from 1 to 9)

The study timeline is illustrated in Fig. 1 and includes two distinct reference points. The first reference timepoint (T0) corresponds to the day of arrival at the EPRU and marks the beginning of the dietary adaptation phase, with subsequent sampling performed at three-day intervals (T3, T6, T9). The second reference timepoint corresponds to the day of foaling. To distinguish it from the arrival-based T0, postpartum timepoints are indicated as T0pp (day of foaling), T3pp (3 days postpartum), and T6pp (6 days postpartum), while pre-foaling samples are indicated as T-6 and T-3. Although two “zero” reference points appear in the graphical representation, they refer to different biological events and were analyzed separately throughout the study.

All mares were clinically healthy. To ensure sample homogeneity, the broodmares originated from the same stable, where they were kept in the same environmental, management, and feeding conditions. The mares were kept at their original facility until reaching 322 ± 5.64 days of gestation (mean ± SD), after which they were transported to EPRU for the experimental phase. The transport covered approximately 140 km and took about 1 h and 30 min.

Upon arrival at EPRU, each mare underwent a thorough clinical examination, including a general physical examination, and haematological and parasitological testing to identify any subclinical diseases or abnormalities. The mares were then housed in individual stalls and monitored daily. Complete daily physical examination included measurements of pulse, temperature, and respiration. After parturition, the broodmares were housed with their foals in the same stalls. Daily examination/assessments were conducted to monitor the mares and foals.

For each mare, a comprehensive record was maintained throughout their stay at the EPRU, documenting the following: weight at arrival; Body Condition Score (BCS) at arrival and departure; parity (primiparous or multiparous); last covering date, expected and actual foaling dates; intake of hay and concentrate feed; and any medical treatments administered. At both arrival and discharge, each mare’s weight and BCS were assessed using standardized methods reported by Bergero (1996) and Henneke et al. (1983). These measurements were critical for calculating the precise hay allocations and monitoring nutritional status and changes over the experimental period. Weight was estimated by measuring thoracic girth using the formula derived from Bergero et al. (1996):

$$\:Weight\:\left(kg\right)=0.0184\:\times\:({thoracic\:girth\:\left(cm\right))}^2-129$$

### Nutritional treatments

The broodmares were fed according to their physiological phase and their body weight, with diets comprising hay and concentrate feeds (Table 2). Hay was provided on the floor (Bordin et al. 2024; Greppi et al. 2024), at 2% (1.8% Dry Matter, DM) of the body weight (NRC 1989; Harris et al. 2017); moreover, its quality, in terms of hygiene, inspection, and nutritional value, was assessed using the methods described by Cavallini et al. (Cavallini et al. 2022). The dietary regimen at the mares’ original facility differed from that at the EPRU, both in the type of forage (alfalfa hay vs. grass hay) and concentrate feed composition. During the initial period at the EPRU, an adaptation to the new feeds was enhanced: a reduced amount of concentrate was provided compared to the original stable, while slowly increasing the new feed as parturition approached (Harris and Shepherd 2021). The concentrate feed reached its maximum allocation during early lactation and was maintained until discharge, to meet the heightened energy demands of milk production (Valle et al. 2017). Animals were individually fed and orts recorded, and the daily ration was divided into three equal portions and administered at 9:00 a.m., 3:00 p.m., and 8:00 p.m. On the day of parturition, mares were offered 250 g (as fed) of wafers composed of grass hay, dehydrated alfalfa, flaked barley, flaked corn, sugar cane molasses, characterized by high palatability (Vinassa et al. 2020). These wafers were dissolved in warm water to encourage hydration and reduce the risk of postpartum constipation. Diets were adequate in terms of providing nutrients according to INRA (Martin-Rosset 2015). According to the time series in Fig. 1, each diet reported in Table 2 was delivered.

**Table 2:** Composition of the ration provided during the feeding trial (as fed). The daily diet (hay plus feed) fed at the original facility and the EPRU during the prepartum, and postpartum phases are provided

		Prepartum ration				Postpartum ration	
	Broodmares	original facility	arrival at EPRU − 6th day	7th − 12th day	13th day - delivery	delivery − 6th day	7th day - discharge
**Alfalfa hay**	All	2% BW					
**Grass hay**	All		2% BW	2% BW	2% BW	2% BW	2% BW
**Concentrate 1ᵃ**	All	2 kg					
**Concentrate 2ᵇ**	550 kg BW		0.3 kg	0.6 kg	0.9 kg	1.6 kg	2.5 kg
	650 kg BW		0.6 kg	1 kg	1.3 kg	1.9 kg	2.7 kg
**Concentrate 3ᶜ**	550 kg BW		0.3 kg	0.4 kg	0.7 kg	1 kg	1 kg
	650 kg BW		0.4 kg	0.7 kg	1 kg	1 kg	1.2 kg

BW, Body Weight

ᵃ Black oats, soft wheat bran, toasted dehulled soy extraction meal, crushed barley, crushed corn. Pelleted: soybean meal, sunflower seed meal, wheat bran, calcium carbonate, dicalcium phosphate, brewer’s yeast, corn, sodium chloride, carob powder, cane and beet molasses, palm oil and fat. Additives (as fed): vitamin A 140,000 IU/kg, vitamin D3 28,000 IU/kg, choline chloride 8,400 mg/kg, niacinamide 350 mg/kg, calcium D-pantothenate 210 mg/kg, vitamin B1 28 mg/kg, vitamin B12 0.28 mg/kg, vitamin B2 84 mg/kg, vitamin B6 28 mg/kg, vitamin E 280 mg/kg, vitamin K3 28 mg/kg

ᵇ Flaked corn, corn, soybean meal, refined vegetable oils and fats (soybean), powdered whey, dried carob. Technological additives (as fed): Kaolin clays, asbestos-free 12.5 g/kg

ᶜ Soybean meal, dehydrated alfalfa (high temperature), dehulled sunflower seed meal, dried sugar beet pulp, whey powder, wheat bran, dried carob, calcium carbonate, toasted soybean, monocalcium phosphate, cane sugar molasses, potato protein, sodium chloride, refined vegetable oils and fats (soy). Nutritional additives (as fed): Vitamin A 140,000 IU/kg, Vitamin D3 1,600 IU/kg, Vitamin E 350 mg/kg, Biotin 200 µg/kg, Choline chloride 160 mg/kg, Iron 75 mg/kg, Iodine 1.1 mg/kg, Cobalt 0.35 mg/kg, Copper 27 mg/kg, Manganese 137 mg/kg, Zinc 145 mg/kg, Selenium 0.70 mg/kg. Technological additives (as fed): Propyl gallate 1.88 mg/kg. Zootechnical additives: Saccharomyces cerevisiae

### Sampling and analysis

**Fig. 2:**
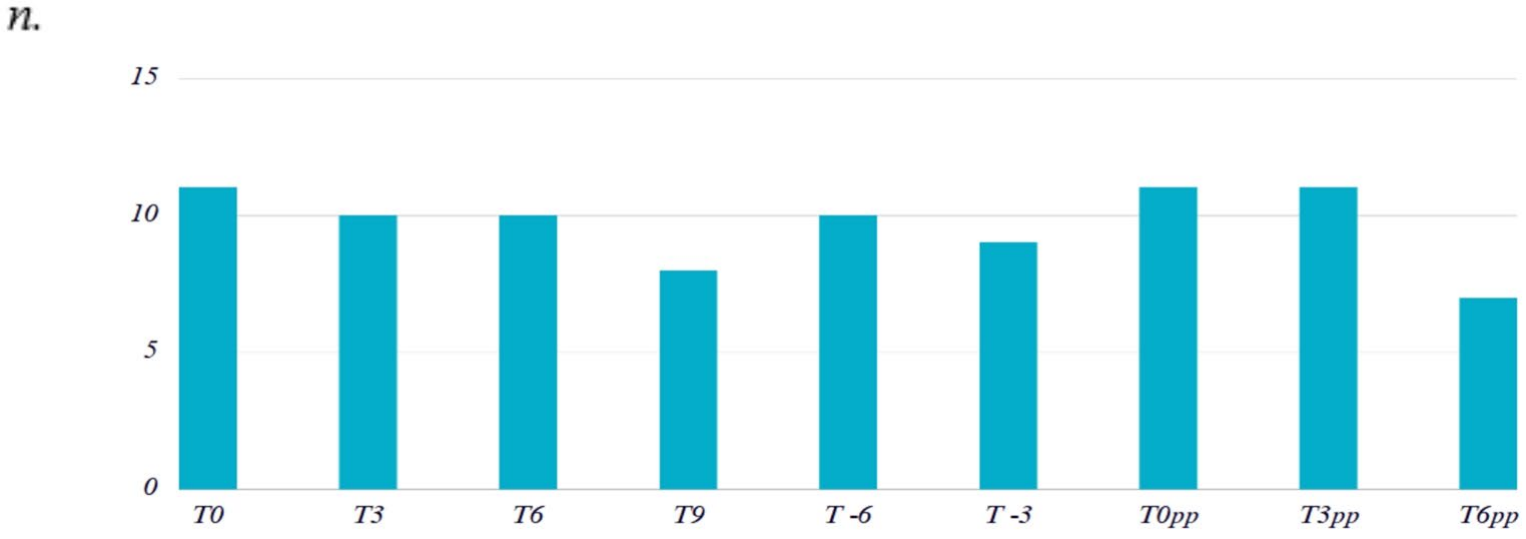
Sample distribution. The average stay was 22.64 ± 7.71 days. A total of eleven samples were collected at time point 0 (T0), ten at T3, ten at T6, eight at T9, ten at T-6, nine at T-3, eleven at T0 postpartum (T0pp) and T3 postpartum (T3pp), and seven at T6 postpartum (T6pp)

At the beginning of the feeding adaptation, feed samples (concentrates and forages) were collected from the EPRU facility and the mares’ original stables. These were identified and stored in airtight bags under refrigeration until analysis. During the study, fecal samples were collected from each broodmare every three days. Sampling was conducted in the early morning, immediately after bedding was refreshed. Due to variations in the timing of foaling, the duration of each mare’s stay at EPRU differed, resulting in variability in the number of samples collected per time point (Fig. 2). The average stay was 22.64 ± 7.71 days. A total of eleven samples were collected at time point 0 (T0), ten at T3, ten at T6, eight at T9, ten at T-6, nine at T-3, eleven at T0 postpartum (T0pp) and T3 postpartum (T3pp), and seven at T6 postpartum (T6pp). Each fecal sample weighed approximately 300 g and was divided into two aliquots. One aliquot was designated for particle size analysis, and the other for nutrient composition and dry matter determination. All aliquots were properly labelled, sealed in airtight containers, and stored at −20 °C until further analysis.

All feeds and fecal samples were analyzed in the laboratories of the Animal Production and Food Safety (SPASA; DIMEVET, University of Bologna). Concerning fecal samples, fecal dry matter content was determined to assess water content, and pH was measured using a pH meter (Seven Multi Mettler-Toledo, Spain). Moreover, fecal samples were analysed for the particle size distribution. Accordingly, each fecal sample was thawed at room temperature and then subjected to wet sieving. Sieves of various sizes were used: 6.70 millimeters, 4.75 millimeters, 3.35 millimeters, 2 millimeters, 1.18 millimeters, 0.6 millimeters, and 0.125 millimeters. First, the sample was weighed and then placed in a modified Ro-Tap machine (Mertens 2003). The machine was activated, and through vibration and the flow of running water, the sample was sieved. Each sieve was then dried and weighed. The difference in weights was used to determine the various fractions characterized by the specific particulate size of the sieve (Raspa et al. 2022b).

Dry matter of feeds and feces samples was evaluated. Samples were dried in a forced-air oven at 65 °C for 72 h according to 950.46 AOAC method. After drying, all samples were ground through a 1-mm screen in a Cyclotec™ 1093 Sample Mill (FOSS Tecator, Hoganas, Sweden).

Determination of nutrients in feeds and fecal samples was performed according to the following procedures: crude protein (CP) was evaluated according to method 984.13 A, using a Kjeldahl nitrogen analyser (Gerhadt Vapodest 50, Gerhardt GmbH, Königswinter, Germany). Starch was determined according to method 920.40 AOAC. Ether extract was measured using a Soxhlet apparatus according to method 920.39 AOAC and ash with method 942.05 AOAC.

For fiber fractions, amylase-treated NDF(Mertens 2002) with addition of sodium sulfite was determined, as well as ADF (AOAC 1990; method 973.18), and ADL (AOAC 1990; method 973.18). Additionally, uNDF determination was performed on fecal samples as described below. The uNDF was calculated by analysing in vitro NDF degradability (dNDF) at 240 h using Tilley and Terry modified technique (U.S. Department of Agriculture 1949; Tilley and Terry 1963; Van Soest et al. 1991). Each fecal sample was analyzed in triplicate in two different in vitro fermentations. Rumen fluid was collected from three Friesian dairy cows housed at the experimental dairy farm, 3 h after the morning feeding. The cows were fed a diet primarily composed of alfalfa hay (35.1% TMR dry matter, DM), wheat hay (11.16% DM), and a cereal mix (48% DM). Fluid was extracted using an oesophageal probe, following the protocol described by Muizelaar et al. (Muizelaar et al. 2020). To minimize saliva contamination, the first 500 mL of rumen fluid was discarded. The collected fluid was immediately transferred into preheated thermoses and transported to the laboratory. Upon arrival, it was strained through a sieve with 1-mm diameter pores to remove larger particulate matter. During this process, bottles containing the rumen fluid were kept in a water bath maintained at 39.5 °C. After filtration, the pH of the rumen fluid was measured using a pH meter (PH20er, VWR, Italy). A 10-mL volume of rumen fluid was inoculated into each Erlenmeyer flask already placed in a heated water bath under CO_2_-positive pressure to ensure anaerobiosis. Each flask contained 40 mL of solution described by Goering and Van Soest(U.S. Department of Agriculture 1949) and 0.50 g of sample. At the end of the fermentation at 240 h, the content of each flask was analyzed to determine NDF content of the residue. Residues were then treated following the procedure described by Palmonari et al.(Palmonari et al. 2016) after 3 h of drying in a forced-air oven (105 °C), and hot weight of the crucibles was recorded. Ash correction was made after incineration of the residue at 495 °C for 3 h, followed by a second crucible hot weighing.

aNDFom disappearance was calculated as:

$$\:dNDF,\:\%aNDFom=1-\left[\frac{{aNDFom}_r-{aNDFom}_b}{{aNDFom}_i}\right]\ast100$$

where aNDFom_r_ is the residual aNDFom, aNDFom_b_ is the blank correction, and aNDFom_i_ represents the initial NDF. All the described terms are expressed in grams.

The uNDF fraction was then calculated using the following equation:

$$\:{uNDF}_{240}\%DM=\left(100-dNDF240h\right)\ast aNDFom/100$$

where aNDFom is the aNDFom content of the sample, uNDF240 is the undegradable NDF residue after 240 h of in vitro fermentation; dNDF240h is the degradability percentage of NDF after 240 h of in vitro fermentation.

After completing the wet chemistry analyses, the obtained reference values were used to calibrate the NIR instrument (TANGO FT-NIR spectrometer, Bruker Optics GmbH, Ettlingen, Germany) according to the procedure described by Buonaiuto et al. (2021). The calibration was performed to adapt and validate the NIR system for future applications in equine feed evaluation. The chemical composition of concentrate feeds and forages determined through wet analyses is reported in Tables 3 and 4.

**Table 3:** Nutritional composition of forages used in the diets of broodmares in late gestation, peripartum and early lactation

% DM	Alfalfa hay	Grass hay
**Ash**	8.78 ± 1.00	9.28 ± 0.32
**Crude Protein**	18.33 ± 2.63	9.73 ± 0.68
**Starch**	2.40 ± 0.60	2.64 ± 0.46
**Ether Extract**	1.81 ± 0.17	1.73 ± 0.16
**aNDFom**	46.08 ± 4.06	56.28 ± 2.87
**ADF**	35.92 ± 3.75	40.51 ± 3.09
**ADL**	7.97 ± 0.77	7.11 ± 1.07
**uNDF**	20.46 ± 3.87	21.23 ± 3.92

DM, Dry Matter

aNDFom, amylase-treated Neutral Detergent Fiber, expressed on organic matter

ADF, Acid Detergent Fiber

ADL, Acid Detergent Lignin

uNDF, undigested Neutral Detergent Fiber

**Table 4:** Nutritional composition of concentrate feeds used in the diets of broodmares in late gestation, peripartum and early lactation

% DM	Concentrate 1	Concentrate 2	Concentrate 3
**Ash**	5.44	7.32	11.79
**Crude Protein**	18.57	11.81	33.45
**Starch**	31.11	45.68	5.61
**Ether Extract**	19.07	5.42	2.58
**aNDFom**	26.89	15.54	25.66
**ADF**	16.24	7.44	17.49
**ADL**	4.58	6.06	5.92
**uNDF**	6.04	2.24	7.16

DM, Dry Matter

aNDFom, amylase-treated Neutral Detergent Fiber, expressed on organic matter

ADF, Acid Detergent Fiber

ADL, Acid Detergent Lignin

uNDF, undigested Neutral Detergent Fiber

### Digestibility calculation

The apparent digestibility of the different components was calculated using uNDF as a marker (Cavallini et al. 2023). The apparent total tract digestibility (TTD) of the dietary components was calculated using uNDF240h as an internal marker (Cavallini et al. 2023). Digestibility was determined for the following variables: organic matter (TTOMD), crude protein (TTCPD), starch (TTStarchD), amylase-treated neutral detergent fiber expressed on organic matter (TTaNDFomD), acid detergent fiber (TTADFD), and potentially digestible neutral detergent fiber (TTpdNDFD).

Potentially digestible NDF (pdNDF) was calculated as the difference between total aNDFom and undigested NDF after 240 h of in vitro fermentation (uNDF240h), according to the following equation:

$$pdNDF=aNDFom-uNDF240h$$

All TTxxxD variables refer to apparent total tract digestibility coefficients calculated using uNDF240h as the internal marker, according to the following formulas:

$$\begin{array}{c}TTOMD,\:\%OM=100-\\\left[\:\frac{dietary\:uNDF,\:\%DM}{fecal\:uNDF,\:\%DM}\ast\frac{fecal\:OM,\%DM}{dietary\:OM,\:\%DM}\right]\ast100\end{array}$$

$$\begin{array}{c}TTCPD,\:\%CP=100-\\\left[\:\frac{dietary\:uNDF,\:\%DM}{fecal\:uNDF,\:\%DM}\ast\frac{fecal\:CP,\%DM}{dietary\:CP,\:\%DM}\right]\ast100\end{array}$$

$$\begin{array}{c}TTstarchD,\:\%starch=100-\\\left[\:\frac{dietary\:uNDF,\:\%DM}{fecal\:uNDF,\:\%DM}\ast\frac{fecal\:starch,\%DM}{dietary\:starch,\:\%DM}\right]\ast100\end{array}$$

$$\begin{array}{c}TTaNDFomD,\:\%aNDFom=100-\\\left[\:\frac{dietary\:uNDF,\:\%DM}{fecal\:uNDF,\:\%DM}\ast\frac{fecal\:aNDFom,\%DM}{dietary\:aNDFom,\:\%DM}\right]\ast100\end{array}$$

$$\begin{array}{c}TTpNDF24hD,\:\%pNDF24h=100-\\\left[\:\frac{dietary\:uNDF,\:\%DM}{fecal\:uNDF,\:\%DM}\ast\frac{fecal\:pNDF24h,\%DM}{dietary\:pNDF24h,\:\%DM}\right]\ast100\end{array}$$

$$\begin{array}{c}\begin{array}{c}TTpNDF240hD,\:\%pNDF240h=100-\\\left[\:\frac{dietary\:uNDF,\:\%DM}{fecal\:uNDF,\:\%DM}\ast\frac{fecal\:pNDF240h,\%DM}{dietary\:pNDF240h,\:\%DM}\right]\ast100\end{array}\end{array}$$

The wafer supplement was not included in the calculations of the results, as it does not affect either the sample from the day of delivery or the subsequent one.

### Statistical analysis

The data obtained were collected in an Excel database (Microsoft 2018) and subsequently analysed using the statistical software JMP Pro v 17 (SAS Institute Inc., Cary, NC, USA). All variables were first evaluated for their distribution and homogeneity (Shapiro-Wilk and Levene tests). A Repeated Measures Mixed Model was then applied to assess the fixed effect of time (sampled time points) on the parameters of interest. Each individual mare was considered the experimental unit and included as a random effect in the model. The residuals from the model were subsequently evaluated to confirm the correctness of the analysis. A significance level of 5% was considered, and the statistical error was expressed as the standard error of the mean (SEM). When the global model was significant, a post hoc test (Tukey) was performed to verify differences between the means obtained.

## Results

Throughout the study, all 11 mares participating in the trial were in good health, and no clinical findings were identified that would compromise their inclusion. The BCS and percent variation in BCS upon arrival and discharge are presented in Table 5. The percentage variation in BCS is also shown. A general decrease in BCS was observed, exceeding 10% in only one case. In seven out of eleven mares observed, a decrease in the BCS value was noted during their stay at the EPRU. This decrease was equivalent to one point in only one case, while in other cases, the score dropped by 0.5 or 0.25.

**Table 5:** Results of recorded BCS of the broodmares during the trial (at the arrival and discharge, and percentage variation calculation)

Broodmare	BCS arrival	BCS discharge	Variation in %
**n.1**	6.5	6	−7.7
**n.2**	7	6.5	−7.1
**n.3**	6.25	6	−4
**n.4**	6.5	6.25	−3.8
**n.5**	6	6	0
**n.6**	7	6	−14.3
**n.7**	6	6	0
**n.8**	6	6	0
**n.9**	6	5.75	−4.2
**n.10**	6.5	6.5	0
**n.11**	6.5	6.25	−3.8

BCS, Body Condition Score

Table 6 describes the intake of concentrate feed and individual nutrients during diet adaptation. Forage intake is not reported because it was standardized at 2% as fed (1.8% DM) of body weight, in fact no refusals were recorded. On the first day (T0), concentrate feed intake in kilograms was significantly higher (1.84 kg on DM basis) compared to subsequent days, decreasing to 0.58 kg DM on the third day, then rising again to 0.89 kg DM on day 6 and 0.99 kg DM on T9 (*p* < 0.01). The OM percentage showed a decrease, dropping from 91.76% on T0 to 90.73% on T9 (*p* < 0.01), while intake in kilograms decreased from 10.74 kg to 10.15 kg, likely due to the reduced amount of feed provided. Protein content also declined (*p* < 0.01), starting at 2.14 kg (18.32% of intake) and stabilizing around 1.20 kg (10.73% of intake) on T9, in line with the reduced total feed concentrate quantity, which influenced protein intake. Intake of starch followed a similar trend (*p* < 0.01), decreasing from the initial 0.80 kg (6.86% of intake) to 0.42 kg (3.89% of intake) on T3, then reaching 0.55 kg (4.95% of intake) on T9, reflecting the reduction in starch-rich feed. The intake of various fiber components, on the other hand, recorded an increase during the adaptation period. The aNDFom rose from an initial 42.98% to a final 53.02% (*p <* 0.01), with intake in kilograms increasing from 5.03 to 5.94 kg (*p* < 0.01), highlighting the shift to more fiber-rich hay. ADF showed a similar trend (*p* < 0.01), rising from 3.84 kg (32.77% of intake) on T0 to 4.20 kg (39.00% of intake) on T3, then progressively decreasing to 4.25 kg (37.96% of intake) on T9. uNDF240 increased (*p* < 0.01) from 19.72% on day 0 to 21.23% on T9, with intake rising from 2.31 to 2.38 kg (*p* < 0.01), reflecting the higher fiber content of the hay. Potentially degradable NDF (pdNDF) followed this trend, increasing from 23.26% on T0 to 33.16% on T3, then decreasing on T6 and T9 (32.11% and 31.82% of intake, respectively; *p* < 0.01), with intake rising from 2.72 kg to 3.57 kg on T3, reaching 3.56 kg on T6 and T9 (*p* < 0.01).

**Table 6:** Intake of concentrate feed and total nutrients as %DM and kg/day during the diet adaptation period. Each column corresponds to a time point, starting from time zero (indicating the day of arrival) and continuing at three-day intervals up to the ninth day

		Adaptation, d					
Intakes	DM	T0	T3	T6	T9	SEM	*p*-value
**Concentrate Feed**	Kg	1.84^A^	0.58^C^	0.89^B^	0.99^B^	0.05	< 0.01
**OM**	%	91.76^A^	90.71^B^	90.72^B^	90.73^B^	0.02	< 0.01
	Kg	10.74^A^	9.78^C^	10.06^B^	10.15^B^	0.24	< 0.01
**CP**	%	18.32^A^	10.41^C^	10.65^B^	10.73^B^	0.05	< 0.01
	Kg	2.14^A^	1.12^C^	1.18^B^	1.20^B^	0.03	< 0.01
**Starch**	%	6.86^A^	3.89^C^	4.73^B^	4.95^B^	0.11	< 0.01
	Kg	0.80^A^	0.42^C^	0.48^B^	0.55^B^	0.02	< 0.01
**aNDFom**	%	42.98^C^	54.36^A^	53.76^A^	53.02^B^	0.15	< 0.01
	Kg	5.03^C^	5.86^B^	5.92^AB^	5.94^A^	0.13	< 0.01
**ADF**	%	32.77^C^	39.00^A^	38.19^B^	37.96^B^	0.12	< 0.01
	Kg	3.84^C^	4.20^B^	4.24^AB^	4.25^A^	0.09	< 0.01
**uNDF240**	%	19.72^B^	21.20^A^	21.23^A^	21.23^A^	0.03	< 0.01
	Kg	2.31^B^	2.29^B^	2.35^A^	2.38^A^	0.05	< 0.01
**pdNDF**	%	23.26^C^	33.16^A^	32.11^B^	31.82^B^	0.13	< 0.01
	Kg	2.72^B^	3.57^A^	3.56^A^	3.56^A^	0.08	< 0.01

DM, Dry Matter

OM, Organic Matter

CP, Crude Protein

aNDFom, amylase-treated Neutral Detergent Fiber, expressed on organic matter

ADF, Acid Detergent Fiber

uNDF240, undigested Neutral Detergent Fiber after 240 h of in vitro fermentation

pdNDF, potentially digestible Neutral Detergent Fiber

^A, B,C^Values within rows with different superscripts differ (*p*<0.05)

In Table 7, the variations in fecal composition and pH throughout the adaptation period are described. The DM content in the feces remained relatively stable, with values ranging from 20.10% on day 0 to 19.14% on T9, showing no significant variations (*p* = 0.67). Conversely, the ash content shows a significant reduction, decreasing from 13.54% on T0 to 10.89% on T3, then increasing to 11.22% on T9 (*p* < 0.01), expressing changes in dietary composition during adaptation. The CP content in the feces decreases significantly, from 9.37% on T0 to 6.21% on T3 and 6.04% on T6, reaching 7.35% on T9 (*p* < 0.01). Starch in the feces decreases from an initial 2.43% to 1.86% on T3 and T6, with a slight increase to 1.93% on T9 (*p* < 0.01). The aNDFom content increases significantly from 66.12% on T0 to 71.77% on T6, stabilizing at 69.99% on T9 (*p* < 0.01), suggesting a higher proportion of insoluble fiber in the feces compared to other nutrients. Similarly, ADF increases from an initial 51.17% to 54.01% on T3, gradually decreasing to 52.98% on T9 (*p* < 0.01). ADL, a component of indigestible fiber, remained stable over time, with values ranging from 16.80% to 17.15% (*p* = 0.85). The uNDF240 shows slight fluctuations, decreasing from 50.33% on T0 to 47.35% on T9, with borderline significance (*p* = 0.05). The pdNDF increases significantly from 15.75% on T0 to 22.29% on T9 (*p* < 0.01). Finally, fecal pH remained stable throughout the adaptation period, with values oscillating between 7.26 and 7.46 during the observed time points, showing no significant variations (*p* = 0.57).

**Table 7:** Fecal content of dry matter and nutrients as a percentage of dry matter during the diet adaptation phase

		Adaptation, d					
Feces	Unit	T0	T3	T6	T9	SEM	*p*-value
**DM**	%	20.10	19.90	19.40	19.14	0.66	0.67
**Ash**	%DM	13.54^A^	10.89^B^	11.03^B^	11.22^B^	0.52	< 0.01
**CP**	%DM	9.37^A^	6.21^BC^	6.04^C^	7.35^B^	0.40	< 0.01
**Starch**	%DM	2.43^A^	1.86^B^	1.86^B^	1.93^AB^	0.08	< 0.01
**aNDFom**	%DM	66.12^B^	71.65^A^	71.77^A^	69.99^A^	1.07	< 0.01
**ADF**	%DM	51.17^B^	54.01^A^	53.93^A^	52.98^AB^	0.64	< 0.01
**ADL**	%DM	16.80	16.86	17.15	16.51	0.57	0.85
**uNDF240**	%DM	50.33^A^	48.49^AB^	49.26^AB^	47.35^B^	0.93	0.05
**pdNDF**	%DM	15.75^B^	23.16^A^	22.51^A^	22.29^A^	0.75	< 0.01
**pH**		7.29	7.46	7.22	7.26	0.16	0.57

DM, Dry Matter

CP, Crude Protein

aNDFom, amylase-treated Neutral Detergent Fiber, expressed on organic matter

ADF, Acid Detergent Fiber

ADL, Acid Detergent Lignin

uNDF240, undigested Neutral Detergent Fiber after 240 h of in vitro fermentation

pdNDF, potentially digestible Neutral Detergent Fiber

^A, B,C^Values within rows with different superscripts differ (*p*<0.05)

**Fig. 3:**
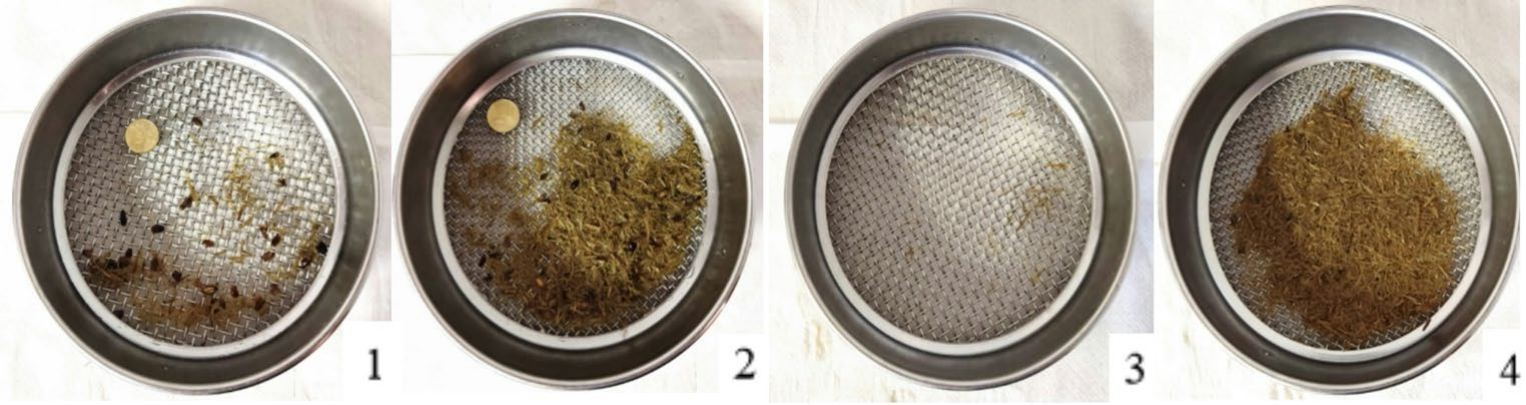
Photographs of sieves showing fecal particle sizes at different time points: 6.7 mm at T0 (1), 4.75 mm at T0 (2), 6.7 mm at T3 (3), and 4.75 mm at T3 (4)

In Table 8, the results of fecal sieving analyses during the adaptation period are shown; each row corresponds to a sieve with different mesh sizes. The values are expressed as a percentage of the total sample. From the table, it is evident that at the arrival time point (T0), the percentage of the two largest sieves is much higher compared to other time points (+ 1.73% at T0 compared to the average of subsequent time points). This result is primarily influenced by the presence of undigested oats at T0, which disappears by T3 (Fig. 3). The percentages of finer sieves increase, albeit not significantly, over the adaptation period, particularly in the 2 mm, 1.18 mm, and 0.6 mm sieves. This indicates the disappearance of oats. Conversely, the percentages of the bottom fraction (< 0.06 mm) remained unchanged.

**Table 8:** Fecal particle size distribution at different time points during the adaptation phase

		Adaptation, d					
Item	Unit	T0	T3	T6	T9	SEM	*p*-value
**6.7 mm**	%	2.58	1.72	0.71	0.12	0.82	0.16
**4.75 mm**	%	5.44^A^	3.26^B^	3.61^B^	3.60^B^	0.39	< 0.01
**3.35 mm**	%	21.74	16.18	23.56	14.49	2.58	0.08
**2 mm**	%	26.55	29.97	26.18	33.11	3.29	0.30
**1.18 mm**	%	16.72	20.32	19.18	19.75	2.56	0.66
**0.6 mm**	%	11.60	14.36	13.31	14.99	1.40	0.29
**< 0.6 mm**	%	14.81	14.18	14.26	13.97	0.39	0.43

^A, B,C^Values within rows with different superscripts differ (*p*<0.05)

The apparent digestibility of the main diet components during the adaptation period are reported in Table 9. The apparent digestibility of OM showed a significant decrease over time, dropping from 62.96% on day 0 to 56.06% on T9 (*p* < 0.01). Similarly, the digestibility of CP also experienced a significant reduction, starting at 79.99% on T0 and decreasing to 73.75% on T3, then rising to 75.49% on T6, before dropping again to 68.89% on T9 (*p* = 0.01). Starch digestibility was initially high, with a value of 86.25% on T0, but it decreased to 79.00% on T3, followed by an increase to 81.99% on T9 (*p* < 0.01). The fibrous components of the diet exhibited no statistical variations in digestibility.

**Table 9:** Total tract apparent digestibility of diet components during the diet adaptation period. Each column corresponds to a time point, starting from time zero (which marks the day of arrival) and continuing at three-day intervals up to the ninth day

	Adaptation, d					
Item, %	T0	T3	T6	T9	SEM	*p*-value
**OM**	62.96^A^	56.96^B^	57.71^B^	56.06^B^	0.63	< 0.01
**CP**	79.99^A^	73.75^AB^	75.49^A^	68.89^B^	2.01	< 0.01
**Starch**	86.25^A^	79.00^C^	82.87^B^	81.99^BC^	0.99	< 0.01
**aNDFom**	39.45	42.31	42.00	41.21	0.81	0.07
**ADF**	38.52	39.37	39.11	37.42	0.83	0.29
**pdNDF**	72.91	69.37	70.17	68.70	1.40	0.15

OM, Organic Matter

CP, Crude Protein

aNDFom, amylase-treated Neutral Detergent Fiber, expressed on organic matter

ADF, Acid Detergent Fiber

pdNDF, potentially digestible Neutral Detergent Fiber

^A, B,C^Values within rows with different superscripts differ (*p*<0.05)

Table 10 describes the intake of feed and individual nutrients during the peripartum period (−15 ± 6.5 days before parturition and + 6.5 ± 2.4 days postpartum). Forage intake is not mentioned because it is standardized at 2% as fed (1.8% DM) of body weight based on the live weight of each mare. The amount of feed consumed increased during the prepartum period, rising from 1.09 kg per day on day − 6 to 1.65 kg on the day of delivery (T0pp), then stabilizing around 1.59 kg by T6pp, with no statistically significant variations (*p* = 0.13). The organic matter percentage remained stable, ranging between 90.71% and 90.73% during the observed period (*p* = 0.59), while intake in kilograms progressively increased from 9.92 kg on day − 6 to 10.75 kg on T3pp, with statistical significance (*p* < 0.01). The percentage of CP showed a slight increase, rising from 10.82% on T-6 to 11.29% on the day of delivery, and then maintaining a similar value in the following days (11.27%; *p* < 0.01). In terms of quantity, CP intake gradually increased from 1.21 kg on T-6 to 1.34 kg on T3pp, with statistically significant differences (*p* < 0.01). The starch content showed a percentage increase, from 5.09% on T-6 to 6.09% on T6pp (*p* = 0.04), while intake in kilograms rose from 0.56 kg to 0.71 kg over the same period, with significance (*p* = 0.03). aNDFom slightly decreased from 52.80% on T-6 to 51.37% on T6pp (*p* = 0.02). However, the intake in kilograms remained constant around 6.11 kg, with minimal but statistically significant variations (*p* < 0.01). ADF showed a percentage decrease from 37.76% on T-6 to 36.63% on T6pp, with statistical significance (*p* = 0.02). In terms of kilograms, ADF decreased from 4.24 kg on T-6 to 4.30 kg on T6pp, with statistical significance (*p* < 0.01). uNDF240 maintained a stable percentage, around 21.22% throughout the period (*p =* 0.16), while intake in kilograms slightly increased from 2.38 kg to 2.49 kg (*p* < 0.01). The pdNDF percentage decreased from 31.58% on T-6 to 29.95% on T3pp, then rose to 39.15% on T6pp (*p* = 0.02). However, the intake in kilograms remained stable around 3.55 kg.

**Table 10:** Concentrate feed and total nutrient intake in %DM and kg/day during the peripartum period. Each column corresponds to a time point, starting six days before foaling. Foaling is designated as time point zero, extending to six days post-foaling with sampling intervals of three days

		Peripartum, d											
Intakes	DM	T-6	T-3		T0pp		T3pp		T6pp		SEM		*p*-value
**Concentrate Feed**	Kg	1.09^B^	1.26^AB^		1.65^A^		1.67^A^		1.59^AB^		0.19		0.13
**OM**	%	90.72	90.71		90.71		90.73		90.71		0.01		0.59
	Kg	9.92^C^	10.05^BC^		10.61^AB^		10.75^A^		10.66^AB^		0.30		< 0.01
**CP**	%	10.82^B^	10.99^AB^		11.29^A^		11.27^A^		11.27^A^		0.12		< 0.01
	Kg	1.21^B^	1.24^AB^		1.27^AB^		1.34^A^		1.31^AB^		0.05		< 0.01
**Starch**	%	5.09^B^	5.42^AB^		6.22^AB^		6.31^A^		6.09^AB^		0.39		0.04
	Kg	0.56^B^	0.60^AB^		0.63^AB^		0.75^A^		0.71^AB^		0.06		0.03
**aNDFom**	%	52.80^A^	52.31^AB^		51.25^B^		51.19^B^		51.37^AB^		0.49		0.02
	Kg	5.93^B^	5.97^AB^		6.05^A^		6.05^A^		6.02^AB^		0.14		< 0.01
**ADF**	%	37.76 ^A^	37.38^AB^		36.53^AB^		36.49^B^		36.63^AB^		0.39		0.02
	Kg	4.24^B^	4.26^AB^		4.28^AB^		4.31^A^		4.30^AB^		0.10		< 0.01
**uNDF240**	%	21.22	21.21		21.23		21.24		21.21		0.02		0.16
	Kg	2.38^B^	2.42^AB^		2.51^A^		2.52^A^		2.49^AB^		0.07		< 0.01
**pdNDF**	%	31.58^A^	31.09^AB^		30.02^AB^		29.95^B^		39.15^AB^		0.49		0.02
	Kg	3.55	3.55		3.54		3.54		3.55		0.18		0.29

DM, Dry Matter

OM, Organic Matter

CP, Crude Protein

aNDFom, amylase-treated Neutral Detergent Fiber, expressed on organic matter

ADF, Acid Detergent Fiber

uNDF240, undigested Neutral Detergent Fiber after 240 h of in vitro fermentation

pdNDF, potentially digestible Neutral Detergent Fiber

^A, B,C^Values within rows with different superscripts differ (*p*<0.05)

In Table 11, the variations in fecal composition and pH during the entire peripartum period are described. Two mares developed clinical signs consistent with postpartum constipation at foaling. Due to the potential alteration of gastrointestinal function associated with this condition, all fecal samples collected from these two mares throughout the study period were excluded from the fecal composition and digestibility analyses. The results presented below therefore refer to the remaining eleven mares. The DM content in feces shows significant variations during the peripartum period, increasing from 20.34% on T-6 to 22.11% on the day of delivery (T0pp) and then stabilizing around 20.28% on T6pp (*p* = 0.02). Ash content does not show significant changes, remaining stable around 10.80% on T-6 and 11.61% on T6pp (*p* = 0.10). CP in feces exhibits a slight increase around the day of delivery, rising from 6.42% on T-6 to 7.3% on T0pp, then decreasing to 6.13% on T3pp and stabilizing at 6.47% on T6pp (*p* = 0.05). Starch in feces increases from 1.91% on T-6 to 2.30% on the day of delivery, then decreases again to 1.87% on T6pp, without statistical significance (*p* = 0.32). The aNDFom shows a decrease from 71.74% on T-6 to 68.09% on the day of delivery, followed by an increase to 71.23% on T3 and 71.51% on T6 (*p* < 0.01). This trend reflects a fluctuation in the proportion of insoluble fiber in feces. ADF exhibits a similar trend, decreasing from 54.10% on T-6 to 52.05% on T0pp and then slightly rising to 53.56% on T6, without statistical significance (*p* = 0.08). ADL remained relatively stable during the period considered, with values ranging from 16.59% to 17.71%, showing no significant changes (*p* = 0.95). The uNDF240 shows a slight reduction, decreasing from 48.75% on T-6 to 46.90% on T0pp and remaining around 49.11% on T6pp (*p* = 0.02). The pdNDF fluctuates between 22.93% on T-6 and 22.45% on T6pp, without significant changes (*p* = 0.11). Fecal pH underwent a decrease, dropping from 7.38 on T-6 to 6.28 on the day of delivery, then rising to 6.73 on T6pp, with statistical significance (*p* = 0.02).

**Table 11:** Fecal content of dry matter and nutrients as a percentage of DM during the peripartum period. Each column corresponds to a time point, starting from six days before parturition. Parturition corresponds to time point zero, extending to six days after parturition with sampling intervals of three days

		Peripartum, d													
Feces	Unit	T-6		T-3		T0pp		T3pp		T6pp		SEM		*p*-value	
**DM**	%DM	20.34^AB^		19.42^B^		22.11^A^		20.82^AB^		20.28^AB^		0.62		0.02	
**Ash**	%DM	10.80		11.44		12.45		11.45		11.61		0.53		0.10	
**CP**	%DM	6.42^AB^		6.31^AB^		7.30^A^		6.13^B^		6.47^AB^		0.44		0.05	
**Starch**	%DM	1.91		1.81		2.30		2.20		1.87		0.07		0.32	
**aNDFom**	%DM	71.74^A^		70.96^AB^		68.09^B^		71.23^A^		71.51^A^		1.02		< 0.01	
**ADF**	%DM	54.10		53.53		52.05		53.65		53.56		0.78		0.08	
**ADL**	%DM	17.14		16.85		17.18		17.71		16.59		0.55		0.95	
**uNDF240**	%DM	48.75^AB^		47.64^AB^		46.90^B^		49.19^A^		49.11^AB^		0.89		0.02	
**pdNDF**	%DM	22.93		23.43		21.13		22.03		22.45		0.73		0.11	
**pH**		7.38^A^		7.09^AB^		6.28^B^		6.97^AB^		6.73^AB^		0.23		0.02	

DM, Dry Matter

CP, Crude Protein

aNDFom, amylase-treated Neutral Detergent Fiber, expressed on organic matter

ADF, Acid Detergent Fiber

ADL, Acid Detergent Lignin

uNDF240, undigested Neutral Detergent Fiber after 240 h of in vitro fermentation

pdNDF, potentially digestible Neutral Detergent Fiber

^A, B,C^Values within rows with different superscripts differ (*p*<0.05)

Regarding the results observed during the peripartum period, the fecal granulometry did not undergo significant alterations (Table 12).

**Table 12:** Fecal particle size distribution at different time points during the peripartum phase

		Peripartum, d						
Item	Unit	T-6	T-3	T0pp	T3pp	T6pp	SEM	*p*-value
6.7 mm	%	0.01	0.05	0.34	0.51	0.71	0.46	0.66
4.75 mm	%	3.15	3.50	3.68	2.33	3.20	1.15	0.72
3.35 mm	%	17.06	15.81	21.46	20.85	25.78	6.35	0.67
2 mm	%	32.45	35.59	28.60	28.60	25.79	4.67	0.51
1.18 mm	%	21.96	13.98	16.76	19.04	20.06	3.77	0.43
0.6 mm	%	11.87	14.97	14.91	13.82	12.04	2.21	0.55
< 0.6 mm	%	14.11	14.39	14.80	14.85	13.68	0.78	0.53

In Table 13, the apparent digestibility of the main dietary components during the peripartum period is reported. The apparent digestibility of OM shows a slight variation during the peripartum period, ranging from 57.09% on day − 6 to 56.22% on the day of parturition (T0pp), followed by an increase to 57.80% on T3pp and 57.85% on T6pp. However, these variations were not statistically significant (*p* = 0.08). The digestibility of CP exhibits a variable but statistically significant trend, decreasing from 73.33% on T-6 to 69.99% on the day of parturition, then progressively increasing to 75.02% on T6pp (*p* = 0.03).

**Table 13:** Apparent digestibility of diet components during the peripartum period. Each column corresponds to a time point, starting six days before parturition. Parturition corresponds to time point zero, extending to six days post-parturition, with sampling intervals of three days

		Peripartum, d						
Total Tract Digestibility	Unit	T-6	T-3	T0pp	T3pp	T6pp	SEM	*p*-value
**OM**	%OM	57.09	56.5	56.22	57.80	57.85	0.75	0.08
**CP**	%CP	73.33^AB^	73.61^AB^	69.99^B^	76.36^A^	75.02^AB^	2.15	0.03
**Starch**	%starch	80.58^C^	82.82^BC^	84.18^ABC^	87.15^A^	86.72^AB^	1.13	< 0.01
**aNDFom**	%NDF	41.76	40.62	40.24	39.83	39.87	1.06	0.45
**ADF**	%ADF	38.61	37.18	36.10	36.38	36.84	1.21	0.42
**pdNDF**	%pdNDF	69.30	67.84	68.53	68.06	67.95	1.49	0.32

OM, Organic Matter

CP, Crude Protein

aNDFom, amylase-treated Neutral Detergent Fiber, expressed on organic matter

ADF, Acid Detergent Fiber

pdNDF, potentially digestible Neutral Detergent Fiber

^A, B,C^Values within rows with different superscripts differ (*p*<0.05)

Starch digestibility varies significantly, rising from 80.58% on T-6 to 82.82% on T-3, with a further increase to 84.18% on the day of parturition. Postpartum, starch digestibility continues to increase, reaching 87.15% on T3pp and 86.72% on T6pp (*p <* 0.01). The fibrous components of the diet show no statistical variability in digestibility.

## Discussion

The present study focused on understanding the digestive and nutritional dynamics of broodmares during the critical phases of late pregnancy and early lactation. Specifically, the research aimed to monitor changes in fiber digestibility and other dietary nutrients, evaluate the effectiveness of a dietary adaptation protocol, and estimate digestibility using uNDF as an intrinsic marker in horses. These objectives were explored to provide more detailed insights into the physiological adaptations occurring during this period and to contribute to the optimization of nutritional strategies for broodmares, ultimately improving their health and the well-being of their foals. The findings of this study offer valuable implications for equine nutrition management, which are discussed in detail below.

The health status of the mares included in the study was optimal. In seven out of eleven observed mares, a decrease in BCS was noted during their stay at the EPRU. This decrease was equivalent to one point in only one case, while in the others, the score decreased by 0.5 or 0.25. This reduction was indicative of slight weight loss, attributable to the metabolic effort associated with foaling and the onset of lactation (Henneke et al. 1984). The other four mares showed no changes. Literature describes a differentiation in the trend of BCS during late pregnancy and lactation, depending on its value in the early stages of gestation. Mares with a score higher than 7.0 tend to experience a decrease, those with a score between 5.0 and 6.5 may show either a decrease or an increase, and those with a score below 5.0 generally show an increase (Henneke et al. 1984). Since all the mares in our experiment arrived with a BCS between 6.0 and 7.0 and experienced either a slight or no decrease in body condition, we can affirm that the results align with those reported in the literature.

Regarding the forages used in the feeding trial (Table 3), the substantial differences between these two types of forage were due to their different nature; original facility hay was alfalfa hay, while the hay from the EPRU was grass hay (first cut or fallow). The protein content was, in fact, twice as high in alfalfa hay, whereas grass hay had higher levels of NDF, ADF, and uNDF. Regarding the lipid component, there were no differences between the two forages, while the starch content was slightly higher in the hay from the EPRU stable. All these characteristics are consistent with the qualities of good alfalfa and grass forage and all hay samples were free of foreign bodies and undesirable species (Cavallini et al. 2022). The equine digestive system is uniquely adapted for fiber digestion, with the large intestine, particularly the cecum and colon, playing a key role in fermenting undigested carbohydrates into volatile fatty acids (VFAs), which serve as a primary energy source (Santos et al. 2011; Martin-Rosset 2015). Proper fiber digestion and water intake are critical for maintaining gastrointestinal health, as disorders such as colon impaction, a common peri-partum condition, are often associated with insufficient water intake and can lead to severe complications if left untreated (Sprayberry 2021). A balanced fiber source is critical for equine diets, particularly for broodmares during late pregnancy and early lactation. A high-quality first-cut polyphyte hay is often considered the best choice for horses due to its balanced nutritional profile and fiber content. While alfalfa hay is an excellent feed source, relying exclusively on it to meet fiber requirements can result in excess protein intake and imbalances in the calcium-to-phosphorus ratio. This can overburden the kidneys and liver, making it less suitable as the sole forage source. For this reason, it is generally recommended that alfalfa hay comprise no more than 30% of the total forage intake, as suggested in Geor et al. (2013). At the EPRU, the chosen diet reflects this principle, emphasizing the use of high-quality polyphyte hay to ensure proper nutritional and hygienic standards. This approach provides a more balanced fiber source while reducing the risks associated with excessive protein intake from alfalfa hay alone (Cavallini et al. 2022). This strategic selection of forage not only supports gastrointestinal health but also aligns with the specific needs of broodmares in critical reproductive phases.

The diet was carefully balanced to meet the energy, protein, mineral, and vitamin requirements of broodmares during the gestation and lactation phases. To increase the energy density of the ration, a cereal-based concentrate was selected, ensuring that the starch intake did not exceed the safe limit of 1 g of starch per kg of body weight per meal, as recommended in the literature (Harris and Shepherd 2021). Additionally, a specific balancer was incorporated into the diet to provide high-quality protein and essential nutrients, ensuring a well-rounded nutritional profile tailored to the physiological demands of the mares.

The feed intake was higher on the first day since, in the originating stable, a larger amount of feed was provided compared to the quantities of feed provided during the adaptation phase. The new feeds were gradually increased over the following days. The amount of concentrate administered at T6 was lower than the theoretical requirements outlined in the adaptation protocol. This occurred primarily because there was not enough time to complete a full transition (typically two weeks on average, as noted by Julliand et al. (2001). It is crucial that the transition is gradual because an abrupt dietary change can lead to rapid consumption by horses, increasing the risk for digestive disorders such as dysbiosis and colic (Spadari et al. 2023). Therefore, the diet planned at EPRU aimed to adhere to safe levels of starch consumption and to introduce gradual changes in feed (Raspa et al. 2022b, 2024).

Interestingly, results regarding the fecal analyses showed a higher starch content at T0. As reported in Fig. 3, oats remained undigested in the feces because, being intact kernels, they are resistant to amylase activity (Kienzle et al. 1997). A high content of undigested feed, specifically the starchy fraction of the feed concentrate, leads to various issues: lack of energy extraction from oats for the mare, presence of undigested material reaching the large intestine, which can promote uncontrolled fermentation and dysbiosis, economic loss for the farmer due to the wastage of a relatively costly raw material, and environmental loss of feed with an associated ecological impact (Geor et al. 2013). Studies by Raspa et al. (2020a, b, 2024) have shown that high-starch diets in horses lead to notable changes in gut health. These diets were associated with increased dry matter content in the right dorsal colon and higher organic matter and ash content in multiple intestinal compartments, including the sternal and pelvic flexures, right dorsal colon, and rectum. Additionally, horses on high-starch diets exhibited smaller particle sizes in the digestive content and higher valeric acid concentrations, along with increased levels of volatile fatty acids. These findings highlight the significant impact of diet on gut environment and microbiota composition, with high-starch diets reducing alpha diversity and promoting amylolytic bacteria. From a clinical perspective, this emphasizes the importance of favoring fiber-rich diets over starch-heavy ones to maintain gut health and prevent dysbiosis in horses. Importantly, in the studies discussed above, the observed alterations were primarily localized in intestinal sites often implicated in digestive problems during colic surgeries. In particular, excessive fermentation in the colon caused by cereal overload can lead to colitis and endotoxemia, frequently accompanied by signs of colic (Hudson et al. 2001; Spadari et al. 2023).

In the present study, the decrease in the intake of OM, which is represented by the difference between DM and ash, is also attributable to the reduction in the amount of feed provided. The same reasoning applies to proteins and starch, which decrease because the feeds from the EPRU, when combined, are equivalent in protein and starch content to original facility feed (Table 4). However, the reduction in the amount administered led to a decrease in the total nutrient intake (1.84 kg of original facility feed vs. 0.58 kg of feed from the EPRU).

Accordingly, the amount of starch intake was reduced due to the lower amount of concentrates fed (0.4 kg/horse/day); this is positive during the transition periods from two different diets to avoid digestive disturbances and, as well known, the maximum amount of digestible starch in horses is set to 1 g/kg BW/meal (Harris and Shepherd 2021), value much lower than what was provided in the present study. Unlike the previous components, the intake in kilograms of the different fibrous types increases from the first to the third day and then remained more or less constant (an average increase of 4.90% in NDF, 3.22% in ADF, 1.34% in uNDF, and 2.57% in pdNDF). This increase is due to the fact that, as feed intake decreases and forage intake remained constant (2% as fed/1.8% DM of body weight), the proportion of forage in relation to dry matter increases. Additionally, the type of hay was changed, transitioning from alfalfa hay to first-cut hay, which is richer in grasses and more fibrous, particularly in aNDFom (+ 10.2%) and uNDF (+ 0.78%) compared to the previous one (Table 3). The fiber increase is beneficial. In fact, a substantial supply of long forage, with high fiber content, increases chewing activity and saliva production (Meyer et al. 1975), prolongs the retention time of feed in the stomach (Coenen 1992), and positively influences microbial fermentations (Willard et al. 1977). Indeed, the mean retention time in horses, as studied by Schwarm et al. (Schwarm et al. 2022), is approximately 22 h for the liquid fraction, which is typically richer in the soluble portion characteristic of concentrates, and 25–26 h for the more solid fraction, largely composed of forage.

The DM did not show significant changes throughout the adaptation period. This finding can be explained by the stability of the fecal water-to-dry-matter ratio, despite the observed reduction in feed intake. Under physiological conditions, fecal DM is mainly determined by fecal water content, which is influenced by two primary mechanisms: (i) the water reabsorption capacity of the large intestine, which can be reduced when intestinal motility increases, leading to looser feces, and (ii) the concentration of osmotically active solutes within the fecal mass (Geor et al. 2013).

Importantly, fiber concentration and fiber type represent the dominant dietary factors affecting fecal DM, as they regulate both water-holding capacity and hindgut fermentation dynamics. Structural carbohydrates, particularly NDF, increase fecal bulk and water retention through their physical properties, while the fermentability of the fiber fraction influences the production of soluble fermentation end-products that contribute to osmotic pressure. In contrast, highly fermentable non-structural carbohydrates, especially starch escaping small intestinal digestion, can increase fecal water content indirectly by enhancing osmotic load in the large intestine (Geor et al. 2013). In the present study, despite the progressive increase in dietary fiber intake and the shift toward a more fibrous forage source during the adaptation period, fecal dry matter and fecal consistency remained stable. This finding suggests that the gradual nature of the dietary transition allowed effective physiological adaptation of hindgut motility and fermentation processes, preventing excessive water retention or dehydration of the fecal mass. Therefore, the absence of changes in fecal DM reflects a maintained balance between intestinal transit, microbial fermentation, and water reabsorption, rather than a static dietary fiber supply. Moreover, the lack of variation in fecal dry matter indicates that both motility and the fermentation pattern in the large intestine did not change with dietary variations, which is a very important and positive aspect (Valle et al. 2013). In the feces, starch and CP decrease from time point zero to time point three, in proportion to their reduction in intake (−0.82% and − 5.12% for starch and crude protein, respectively). This suggests that feces serve as a direct marker of dietary starch and protein content. It is well-known that horses have a limited capacity for starch digestion, which is subject to a saturation effect (Geor et al. 2013). The higher starch presence at the initial time points is due to the greater amount of undigested oats in the feces during the early time points. Ash content significantly decreases from the first to the second time point (−2.65%), despite the feed and hay provided at the EPRU stable being richer in ash. This result may be attributed to two potential causes: the first possibility is that the total intake of ash was higher at the original facility due to a greater overall feed intake, while the second possibility is that some of the minerals ingested during the adaptation period were more digestible (Sjaastad et al. 2010). Fibrous components, particularly aNDFom and ADF, increased from the first to the third day (+ 5.53% and + 2.84% for NDF and ADF, respectively) and then stabilized. This is caused by an increased intake of these nutrients (Table 6), driven by a proportional increase in forage intake relative to the total diet. The only fibrous fraction that remained more or less unchanged in the feces is ADL. Fecal pH analysis was performed since it is an important indicator of gut health (Berg et al. 2005). In our study, fecal pH did not exhibit significant changes during the adaptation period. Combined with the dry matter data, this confirms that there were no changes in large intestine fermentation. A potential increase in fermentation, particularly of the starchy components, would have acidified the feces.

Regarding organic matter, proteins, and starch, digestibility decreased from T0 onward (−2.30%, −3.71%, and − 1.41% respectively for organic matter, proteins, and starch). Theoretically, the digestibility of these components should have increased as they were administered in smaller quantities. An additional explanation for the observed reduction in organic matter, starch, and crude protein digestibility may be related to the concurrent increase in dietary fiber. Diets richer in structural carbohydrates are known to influence gastrointestinal transit dynamics in horses. An increased fiber proportion tends to enhance retention time in the hindgut, favouring microbial fermentation of fibrous fractions, while simultaneously accelerating passage through the small intestine. As a consequence, enzymatic digestion of starch and proteins in the foregut may be partially reduced due to shorter residence time, resulting in lower apparent digestibility of these nutrients (Geor et al. 2013). Conversely, the prolonged retention of fibrous components in the cecum and colon may explain the stability or slight improvement observed in fiber digestibility, as microorganisms are provided with more time to degrade structural carbohydrates. This fiber digestibility decrease could be attributed to a change in both forage and feed, leading to a shift in the nature of the components. The change in diet required the adaptation of both enzymatic activity (gastric, duodenal, biliary, and pancreatic) and the cecal and colonic microflora, resulting in an initial loss of digestibility. Two hypotheses could explain this decline:

The time needed for the adaptation to the new diet and for the synthesis of an optimal microflora might be longer than the nine days observed in this study. For instance, first, other studies have estimated adaptation periods of 14 days (Julliand et al. 2001) or even 21 days (Scott et al. 1992). Second, the supplied feed might be less digestible compared to the previous one. Regarding feed composition (Table 4), the starchy portions transitioned from a feed predominantly based on oats to one containing flaked maize, which theoretically has higher digestibility (de Fombelle et al. 2004; Rosenfeld & Austbø, 2009). For this reason, the first hypothesis, that a longer adaptation period was necessary, seems more plausible. As for protein sources, both feeds derived their protein content from soybean meal. In this case, digestibility varies primarily depending on the temperature of soybean toasting and the degree of denaturation during processing. While we cannot evaluate this factor directly, observing that starch digestibility decreased instead of increasing with the change in feed type, we hypothesize that this is also related to adaptation. These considerations could be the focus of future studies designed specifically to address this issue. The digestibility of fibrous fractions remained high and unchanged during the adaptation period. Literature reports various studies on fiber digestibility, with values ranging from 45 to 50% for NDF and 39.9% for ADF (Pagan 1998; Elzinga et al. 2014). Compared to our findings, aNDFom values are slightly higher, while ADF values are almost identical. This consistency suggests that the use of uNDF as an intrinsic marker provides digestibility estimates comparable to those obtained through total fecal collection, although further validation in equine-specific models would be desirable. The use of indigestible fiber fractions as internal markers is conceptually based on the detergent system described by Van Soest (1994), which allows the identification of indigestible fiber pools within plant cell walls. In ruminant nutrition, uNDF has been widely adopted to estimate total tract digestibility and to improve ration formulation (Palmonari et al. 2021). Moreover, the application of internal markers has been proposed as a practical alternative to total fecal collection in horses, particularly under field conditions where complete collection is difficult to implement (Sales 2012). In the present study, uNDF was used to verify the changes in apparent digestibility occurring during the dietary transition and peripartum phases. The digestibility values obtained were consistent with previously published data derived from total fecal collection methods (Pagan 1998; Elzinga et al. 2014), suggesting that uNDF can provide reliable estimates of nutrient utilization in broodmares under clinical conditions.

Despite the observed lower digestibility of starch, proteins, and organic matter, the fecal content of starch, proteins, and dry matter was not elevated (Table 7). Furthermore, the digestibility of fibrous fractions remained high, pH levels did not change, and fecal scores revealed no abnormalities. Thus, although digestibility decreased, no digestive issues were caused.

Over time, the feed intake in kilograms increases because more is provided to meet the mare’s higher nutritional requirements near foaling and during lactation (Henneke et al. 1984). OM increases only in kilograms and not as a percentage of dry matter, as the amount of feed with comparable ash content is raised. Proteins and starch progressively increased along with feed intake, confirming previous observations that feces are a reliable indicator of the quantity of nutrients provided in the diet. Comparing the daily protein requirements outlined by the NRC (2007) − 0.89–1.07 kg/day at the end of gestation and 1.54–1.84 kg/day during lactation (Geor et al. 2013) - with the intakes described in Table 10, we can assert that the daily protein intake was adequate for the successful completion of gestation (+ 0.24 kg/day on average) but slightly insufficient at the beginning of lactation (−0.38 kg/day on average). This value would likely have increased in subsequent days if the nutritional protocol had been followed, as the initial days of lactation are also a period of adaptation to the new diet (see Table 2). The intake of all fibrous fractions decreases proportionally with the increase in concentrate feeds. The pdNDF (%) decreased because, with the increased concentrate intake, the aNDFom quantity decreased while the uNDF remained unchanged. Since pdNDF is derived by subtracting uNDF from NDF, it decreases. There are no reported requirements for mares, or, in general, for horses, regarding these fibrous fractions in the literature. This study could serve as a foundation for future evaluations.

In Table 11, an increase in fecal DM on the day of delivery (+ 2.69% compared to T-3) is highlighted. The increase in dry matter is the main risk factor for postpartum constipation, as it implies greater fecal dehydration, which in most cases is caused by a reduced transit speed within the intestinal lumen. Transit speed, in turn, has been shown to be strongly influenced by the foetus’ abdominal fill and management changes and physiological state alterations (e.g., delivery) as previously reported in the mean retention time (Williams et al. 2014). Another factor that may be associated with the decreased fecal water content is the general dehydration affecting most mares at the time of delivery. Water losses are linked to excessive sweating, milk production, and the production of fluids for lubricating the birth canal. Additionally, many mares, particularly primiparous ones, often stop drinking water just before and immediately after delivery (Friend 2000). During the experiment, two mares were excluded from the study because they developed postpartum constipation. This pathological condition is frequently observed, but its etiopathogenic mechanism is not well understood. Hypothesized causes are related to both pain in the birth canal, which reflects on the neighbouring enteric tract causing reduced motility, and topographical alterations of intestinal segments (dislocations) due to the disappearance of the fetal mass (LeBlanc 2008).

Regarding fecal composition, the main changes on the day of parturition involved CP, fiber fractions, and pH. The significant increase of fecal CP can be attributed to all the physiological changes that occur when the mare gives birth, causing reduced absorption. Indeed, in subsequent time points, the values stabilize to levels similar to those observed before parturition. The increase in fecal proteins on the day of birth could also be due to increased cellular shedding caused by greater stool dryness (an increase in fecal dry matter and a decrease in water content). The various fibrous fractions didn’t behave uniformly: fecal aNDFom decreased significantly on the day of parturition (T0pp) (−2.87% compared to T-3), as did fecal uNDF (−0.73% compared to T-3) and fecal pdNDF. In contrast, fecal ADL remained more or less constant, with a slight but not significant increase on day zero.

The fecal pH underwent significant variations: it progressively became more acidic as parturition approached, reaching the peak of acidity at T0pp (6.28). Subsequently, it became more basic but did not return to pre-parturition levels. This may be caused by the decreased flow rate observed on the day of birth, which leads to greater food retention in the large intestine, promoting fermentation and increasing the activity of intestinal bacteria. This results in the acidification of the intestinal environment and, consequently, the feces (Garber et al. 2020).

During the peripartum period, fecal particle size distribution did not show significant changes (Table 12), particularly between the T − 3 and T3pp timepoints relative to foaling. This stability suggests that the dietary transition and the physiological events associated with parturition did not markedly alter chewing efficiency, digesta comminution, or hindgut fermentation patterns.

A comparison with Raspa et al. (2022b), who reported differences in fecal granulometry between horses fed high-starch and high-fiber diets, supports this interpretation. In that study, diets richer in concentrates were associated with a greater proportion of coarse particles, likely due to faster transit and reduced fermentation. In contrast, the high forage-to-concentrate ratio used in the present study may have contributed to maintaining consistent particle size distribution throughout the peripartum phase. Therefore, the absence of granulometric changes likely reflects the stability of the dietary fiber supply and gastrointestinal transit conditions during this period.

OM digestibility does not appear to show significant alterations throughout the peripartum period. It is worth noting the significant decrease in protein fraction digestibility on the day of parturition; this finding can be associated with the increase in fecal protein percentage (shown in Table 13). This result confirms previous statements regarding the adaptation period. Literature reports an apparent crude protein digestibility of 71–75% (Pagan 1998; Elzinga et al. 2014), so the results in the table align with published data in horses. In their study (Pagan 1998; Elzinga et al. 2014), the “total fecal collection” method was used, supplemented by the Lucas test (which estimates endogenous intestinal losses and allows for the calculation of true digestibility. Interestingly, the obtained data are consistent across both methodologies. Using uNDF involved only a single sample, significantly reducing the workload required for both sampling and analysis, making this type of experiment applicable not only to university and research facilities but also to field operations. The practical advantage of this approach lies in the possibility of estimating apparent digestibility from spot fecal samples, reducing animal handling and labor requirements, and potentially facilitating its application in larger-scale or field-based studies.

Regarding starch, an increase in digestibility was observed at time point T0pp (Table 13), concomitant with a moderate increase in intake (Table 10). However, the amount of starch provided remained below the threshold generally considered capable of overwhelming the digestive capacity of the small intestine. Therefore, it is unlikely that a substantial proportion of undigested starch reached the large intestine. Although enhanced fermentation of residual starch cannot be entirely excluded, this mechanism alone may not fully explain the transient reduction in fecal pH observed at foaling (Table 11).

An alternative explanation may relate to physiological events associated with parturition. A temporary reduction in gastrointestinal motility around foaling could have slowed the passage of fermentable substrates, including both starch and fiber, prolonging microbial activity and volatile fatty acid (VFA) production in the hindgut. Even though apparent fiber digestibility did not significantly change during this period (Table 13), localized alterations in fermentation dynamics or VFA absorption may have occurred. Previous studies have shown that diet-induced increases in fermentation can reduce hindgut pH (Raspa et al. 2022b); however, in the present study, the fact that fecal pH differed significantly only at T0pp, and only when compared with T-6 (Table 11), supports the hypothesis of a short-term physiological effect rather than a diet-driven alteration in fermentation pattern. Moreover, systemic adjustments occurring at foaling, including fluid shifts, transient dehydration, and possible modifications in intestinal perfusion or buffering capacity, may also have contributed to the temporary acidification observed.

Fiber digestion does not seem to be influenced by the time of parturition. Slight negative variations in ADF and aNDFom and slight positive variations in pdNDF are observed but are not statistically significant. Nevertheless, the recorded apparent fiber digestibility values are high and comparable to those reported by Pagan (1998), which are 45% for NDF and 39.9% for ADF. Moreover, digestibility was not compromised by the acidification mentioned above.

The study has some limitations. The sampling schedule varied slightly among individuals (ranging from 7 to 11), potentially affecting data consistency across study phases. The small group of 11 broodmares provides valuable insights but may not fully represent larger populations or other breeds. Additionally, the observation period was relatively short, possibly limiting the ability to observe longer-term effects of dietary adaptations and the use of uNDF as a digestibility marker. Although digestibility estimates obtained using uNDF as an intrinsic marker were comparable to values reported in the literature, further research is needed to fully validate this approach as a reliable alternative to total fecal collection in equine nutrition studies.

## Conclusions

Under the conditions of this study, the dietary management implemented at the EPRU was associated with stable digestive parameters during late gestation and early lactation. However, given the absence of a control group, no conclusions can be drawn regarding its comparative advantage over other feeding strategies.These findings should therefore be interpreted as descriptive of physiological adaptations occurring under the applied management conditions rather than as evidence supporting a specific feeding strategy. During the adaptation phase, apparent total tract digestibility of organic matter, crude protein, and starch showed modest reductions, whereas fiber digestibility remained largely stable and within the ranges reported in the literature for healthy horses. Overall, these findings indicate no evidence of marked impairment in nutrient utilization during the late-gestation dietary transition. Around parturition, changes in fecal dry matter and fecal pH suggested transient alterations in gastrointestinal water balance and hindgut fermentation dynamics, particularly on the day of foaling, without progression to overt clinical disease. The increase in fecal dry matter observed at parturition may indicate a temporary increased susceptibility to postpartum constipation.

Apparent starch digestibility increased during the peripartum period, concomitant with a reduction in fecal pH. This variation may reflect short-term changes in gastrointestinal transit and microbial activity around parturition, but it should also be interpreted in light of the progressive increase in starch intake during this phase, which may have contributed to both digestibility dynamics and fecal pH variation. Regarding the use of uNDF as an internal marker, digestibility estimates obtained in this study were consistent with published values derived from total fecal collection approaches. This supports the applicability of uNDF as a practical and non-invasive tool for estimating nutrient digestibility in equine nutrition research. Overall, the present study emphasizes the importance of gradual dietary adaptation in broodmares and supports the use of uNDF-based calculations to facilitate digestibility assessment under clinical and field conditions, contributing to the optimization of nutritional strategies during late pregnancy and early lactation, while highlighting the need for further validation in equine-specific models.

## Data Availability

The datasets generated and analysed during the current study are available from the corresponding author upon reasonable request.
